# Sirtuin1 protects endothelial Caveolin-1 expression and preserves endothelial function via suppressing miR-204 and endoplasmic reticulum stress

**DOI:** 10.1038/srep42265

**Published:** 2017-02-09

**Authors:** M. Kassan, A. Vikram, Y. R. Kim, Q. Li, A. Kassan, H. H. Patel, S. Kumar, M. Gabani, J. Liu, J. S. Jacobs, K. Irani

**Affiliations:** 1 Cardiovascular Division, Department of Internal Medicine, Abboud Cardiovascular Research Center, University of Iowa Carver College of Medicine, Iowa City, USA; 2 University of California, San Diego, USA; 3 VA San Diego Healthcare System, University of California, San Diego, USA

## Abstract

Sirtuin1 (Sirt1) is a class III histone deacetylase that regulates a variety of physiological processes, including endothelial function. Caveolin1 (Cav1) is also an important determinant of endothelial function. We asked if Sirt1 governs endothelial Cav1 and endothelial function by regulating miR-204 expression and endoplasmic reticulum (ER) stress. Knockdown of Sirt1 in endothelial cells, and *in vivo* deletion of endothelial Sirt1, induced endothelial ER stress and miR-204 expression, reduced Cav1, and impaired endothelium-dependent vasorelaxation. All of these effects were reversed by a miR-204 inhibitor (miR-204 I) or with overexpression of Cav1. A miR-204 mimic (miR-204 M) decreased Cav1 in endothelial cells. In addition, high-fat diet (HFD) feeding induced vascular miR-204 and reduced endothelial Cav1. MiR-204-I protected against HFD-induced downregulation of endothelial Cav1. Moreover, pharmacologic induction of ER stress with tunicamycin downregulated endothelial Cav1 and impaired endothelium-dependent vasorelaxation that was rescued by overexpressing Cav1. In conclusion, Sirt1 preserves Cav1-dependent endothelial function by mitigating miR-204-mediated vascular ER stress.

Sirt1 belongs to the sirtuin family of nicotinamide adenine dinucleotide (NAD+)-dependent protein deacetylases, whose activation protects against cardiovascular diseases^1^,^2^. Sirt1 promotes endothelium-dependent vascular relaxation by activating endothelial nitric oxide synthase^3^. Caveolin 1 (Cav1) is expressed in the plasma membrane caveolae of the endothelial cells and has an important role in the regulation of vascular function^4^. Additionally, a recent report indicates that Cav1 is a direct binding partner of Sirt1 in mouse embryonic fibroblasts^5^.

MicroRNAs (miRNAs) are short non-coding RNAs that regulate target gene expression in a post-transcriptional manner. More than 16 miRNAs modulate Sirt1 expression^6^. MicroRNA regulation of Sirt1 might affect a wide variety of pathways in humans, from metabolic diseases such as diabetes to cardiovascular diseases and cancer^6^. Vascular miR-204 impaired endothelial dependent vasorelaxation by downregulation of Sirt1^7^.

Several miRNAs have been reported to regulate the ER stress response, either through specific targets or through mechanisms that are yet unclear^8^. Recently, it has been shown that miR-204 is implicated in the ER stress responsive gene modulation and apoptosis in cancer^9^,^10^. Additionally, miR-211, which shares an almost identical sequence with miR-204, has been reported to be induced in a PERK-dependent manner^11^. Moreover, ER stress reduces Cav1^12^ and overexpression of Cav1 mitigates ER stress in prostate cancer cells^13^. In addition, in renal cancer cells Cav1 has been shown to be a target of miR-204^14^. However, the relation between miR-204 and Cav1 in endothelial cells is yet to be determined.

Given this background and the known role of Sirt1 in endothelium-dependent vascular function, we explored if lack of Sirt1 downregulates Cav1 and produces endothelial dysfunction through induction of ER stress and miR-204.

## Results

### Endothelial Sirt1 protects Cav1 expression by downregulating miR-204

**Figure 1:**
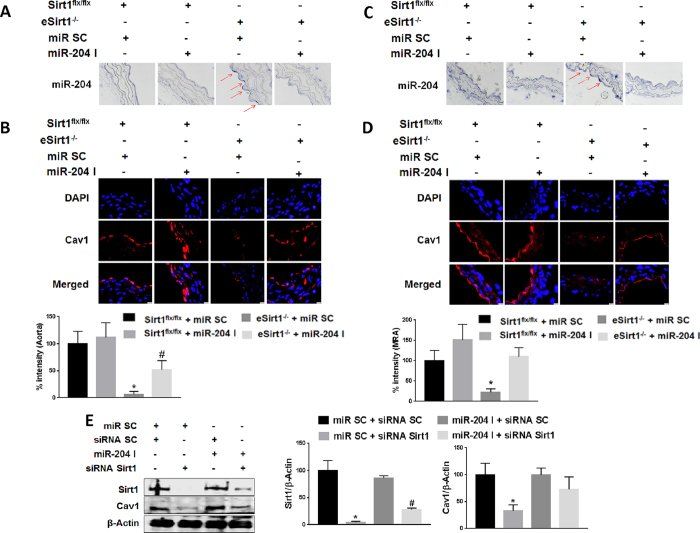
Sirt1 supports endothelial Cav1 expression via downregulation of miR-204. *In situ* hybridization for miR-204 **(A,C)** and immunofluorescence for Cav1 (**B,D**) in thoracic aorta **(A,B)** and mesenteric resistance arteries (MRA) (**C,D**) of eSirt1^−/−^ and Sirt1^flx/flx^ mice systemically infused with miR-204 inhibitor oligonucleotide (miR-204 I) or scrambled control oligonucleotide (miR SC). Quantification of endothelial Cav1 is shown below immunofluorescence images. Red arrows point to miR-204. *p < 0.05 between eSirt1^−/−^ + miR SC vs. Sirt1^flx/flx^ + miR SC, Sirt1^flx/flx^ + miR-204 I and eSirt1^−/−^ + miR-204 I. ^#^p < 0.05 between eSirt1^−/−^ + miR-204 I vs. Sirt1^flx/flx^ + miR SC, Sirt1^flx/flx^ + miR-204 I**. (E)** Immunoblots for Sirt1 and Cav1 in HUVEC transfected with siRNA for Sirt1 or scrambled control siRNA (siRNA SC), and miR-204 I or miR SC. Data is representative of three independent experiments and densitometric quantification shown on right. *p < 0.05 between miR SC + siRNA Sirt1 vs. miR SC + siRNA SC, miR-204 I + siRNA SC and miR-204 I + siRNA Sirt1. ^#^p < 0.05 between miR-204 I + siRNA Sirt1 vs. miR SC + siRNA SC, miR-204 I + siRNA SC.

To study the effect of Sirt1 on vascular Cav1 and miR-204, we generated mice conditionally lacking endothelial Sirt1 (eSirt1^−/−^). Endothelial miR-204 was upregulated, and Cav1 is downregulated, in both thoracic aortas and mesenteric resistance arteries of eSirt1^−/−^ mice (Fig. 1A–D). To determine if down-regulation of Cav1 in eSirt1^−/−^ mice is due to miR-204, miR-204 inhibitor (miR-204 I) was transfected ex *vivo* into both thoracic aortas and mesenteric resistance arteries of eSirt1^−/−^ mice, resulting in suppression of miR-204 expression (Fig. 1A,C). MiR-204 I rescued Cav1 (Fig. 1B,D).

We also determined if Sirt1 regulates Cav1 expression in human umbilical vein endothelial cells (HUVECs) *in vitro*. SiRNA-mediated knockdown of Sirt1 downregulated Cav1 in HUVECs (Fig. 1E). Further, miR-204 I rescued Cav1 expression (Fig. 1E). Taken together, these findings show that Sirt1 protects endothelial Cav1 expression by suppressing miR-204.

### MiR-204 downregulates endothelial Cav1

**Figure 2:**
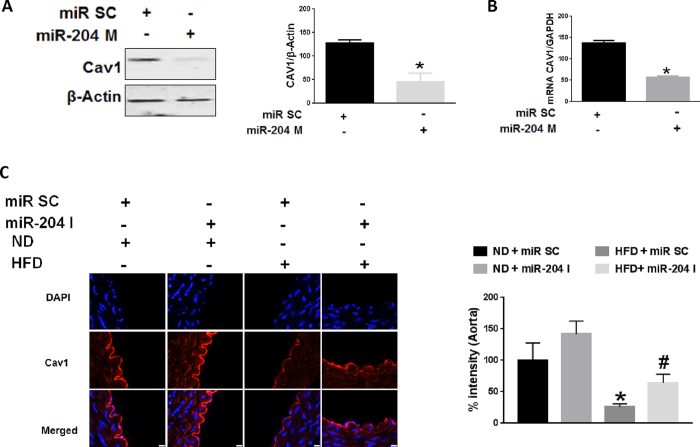
MiR-204 downregulates endothelial Cav1. Immunoblot with quantification on right **(A)** and qPCR **(B)** for Cav1 in HUVECs transfected with miR-204 mimic (miR-204 M) or miR SC. Data is representative of three independent experiments. *p < 0.05 between miR-204 M vs. miR SC **(C)** Immunofluorescence with quantification on right for Cav1 in aortas of mice subjected to high fat diet-feeding for 12 weeks (HFD), and systemically infused with miR-204 I or miR SC. ND: normal chow diet. *p < 0.05 between HFD + miR SC vs. ND + miR SC, ND + miR-204 I and HFD + miR-204 I. ^#^p < 0.05 between HFD + miR-204 I vs. ND + miR SC and ND + miR-204 I.

We next determined the effect of miR-204 on endothelial Cav1 expression, independent of Sirt1. HUVECs were transfected with a miR-204 mimic oligonucleotide (miR-204 M). MiR-204 mimic (miR-204 M) suppressed Cav1 protein (Fig. 2A) and mRNA (Fig. 2B), suggesting that miR-204 targets Cav1 at the post-transcriptional level. We also asked if miR-204 is responsible for Cav1 downregulation in a pathophysiological model of endothelial dysfunction. High fat diet (HFD) feeding of C57Bl/6 mice leads to obesity and endothelial dysfunction, bu not atherosclerosis. Immunofluorescence for Cav1 in thoracic aortas of C57Bl/6 mice on a HFD demonstrated reduction of endothelial Cav1 (Fig. 2C). In addition, systemic infusion of miR-204 I in HFD-fed mice rescued endothelial Cav1 expression (Fig. 2C). These finding underscore the important role of miR-204 in downregulating endothelial Cav1 in a pathophysiological model of endothelial dysfunction.

### Cav1 rescues impaired endothelium-dependent vasorelaxation due to lack of Sirt1

**Figure 3:**
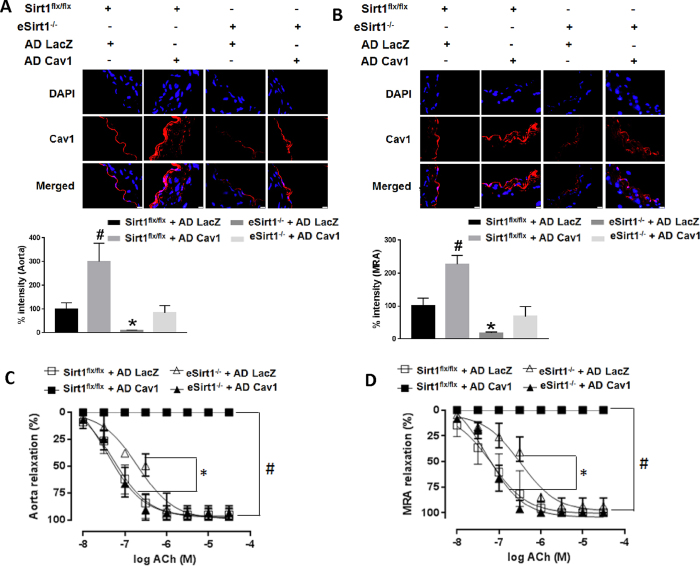
Cav1 rescues endothelial dysfunction due to lack of Sirt1. Immunofluorescence for Cav1 in aortas **(A)** and mesenteric resistance arteries (MRA) **(B)** of mice with conditional deletion of endothelial Sirt1 (eSirt1^−/−^) and control Sirt1^flx/flx^ mice, infected *ex vivo* with adenovirus expressing Cav1 (AD Cav1) or control adenovirus expressing LacZ (AD LacZ). Quantification of endothelial Cav1 is shown below immunofluorescence images. *p < 0.05 between eSirt1^−/−^ + AD LacZ vs. Sirt1^flx/flx^ + AD LacZ, Sirt1^flx/flx^ + AD Cav1 and eSirt1^−/−^ + AD Cav1. ^#^p < 0.05 between Sirt1^flx/flx^ + AD Cav1 vs. Sirt1^flx/flx^ + AD LacZ, and eSirt1^−/−^ + AD Cav1. Endothelium-dependent relaxation in thoracic aortas (**C**) and MRA **(D)** of eSirt1^−/−^ and Sirt1^flx/flx^ mice, infected *ex vivo* with AD Cav1 or AD LacZ. *p < 0.05 between eSirt1^−/−^ + AD LacZ vs. Sirt1^flx/flx^ + AD LacZ, Sirt1^flx/flx^ + AD Cav1 and eSirt1^−/−^ + AD Cav1. ^#^p < 0.05 between Sirt1^flx/flx^ + AD Cav1 vs. eSirt1^−/−^ + AD LacZ, Sirt1^flx/flx^ + AD LacZ, or eSirt1^−/−^ + AD Cav1.

We asked if downregulation of Cav1 observed in eSirt1^−/−^ mice is responsible for impaired endothelium-dependent vasorelaxation in these mice. To answer this question, we reconstituted Cav1 *ex vivo* in aortas and MRA of eSirt1^−/−^ mice using a recombinant adenovirus (AD Cav1) (Fig. 3A,B). Endothelium-dependent vasorelaxation was impaired in aortas and MRA of eSirt1^−/−^ mice compared to aortas and MRA from control Sirt1^flx/flx^ mice (Fig. 3C,D). Importantly, reconstitution of Cav1 restored normal endothelium-dependent vasorelaxation in eSirt1^−/−^ mice (Fig. 3C,D). However, Cav1 overexpression in vessels from Sirt1^flx/flx^ mice in which endothelial function is normal, paradoxically led to marked impairment of endothelium-dependent vasorelaxation (Fig. 3C,D). These findings underscore that normal endothelial function maintained by Sirt1 is mediated through Cav1, but also highlight that excessive Cav1 expression in the vasculature is deleterious for endothelial function.

### ER stress downregulates endothelial Cav1 through miR-204

**Figure 4:**
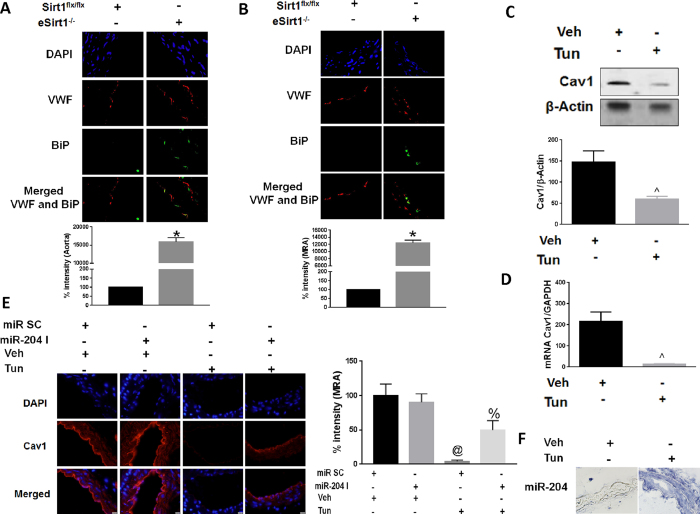
ER stress downregulates endothelial Cav1 through miR-204. Immunofluorescence for ER stress marker BiP in thoracic aorta **(A)** and MRA **(B)** from eSirt1^−/−^ and Sirt1^flx/flx^ mice. *p < 0.05 between eSirt1^−/−^ vs. Sirt1^flx/flx^. Quantification of endothelial BiP is shown at bottom. Immunoblots **(C)** for Cav1 qPCR **(D)** for Cav1 in HUVECs treated with Tunicamycin (Tun) or vehicle (Veh). Quantification of immunoblots is shown at bottom. Data is representative of three independent experiments. ^p < 0.05 between Tun vs. Veh. (**E**) Immunofluorescence for Cav1 in MRA of C57Bl/6 mice treated with tunicamycin (Tun) for 2 weeks with systemic infusion of miR-204 I or miR SC. @p < 0.05 between miR SC + Tun vs. miR SC + Veh, miR-204 I + Veh and miR-204 I + Tun. %p < 0.05 between miR-204 I + Tun vs. miR SC + Veh and miR-204 I + Veh. (**F**) *In situ* hybridization for miR-204 in MRA of C57Bl/6 mice treated with tunicamycin for 2 weeks when compared to mice treated with vehicle.

MiR-204 has been implicated in promoting endoplasmic reticulum stress in other tissues^8^,^9^,^15^. Because ER stress is associated with endothelial dysfunction^16^, we explored the relationship between endothelial Cav1, miR-204, and ER stress. BiP, a marker of ER stress, was induced in aortas and MRA of eSirt1^−/−^ mice (Fig. 4A,B). In addition, the protein glycosylation inhibitor tunicamycin (Tun), a pharmacologic stimulant of ER stress, downregulated Cav1 in HUVECs and in MRA of mice *in vivo* (Fig. 4C,D) while increasing miR-204 expression in MRA (Fig. 4F). To establish a causal role for miR-204 in ER stress-induced downregulation of vascular Cav1, we systemically infused mice with miR-204 I. Tunicamycin-induced downregulation of vascular Cav1 was partially abrogated in MRA of mice that received miR-204 I compared to those that didn’t (Fig. 4E). These data demonstrate that upregulation of vascular miR-204 is responsible for ER stress-induced downregulation of Cav1.

### Cav1 protects against ER stress-induced endothelial dysfunction

**Figure 5:**
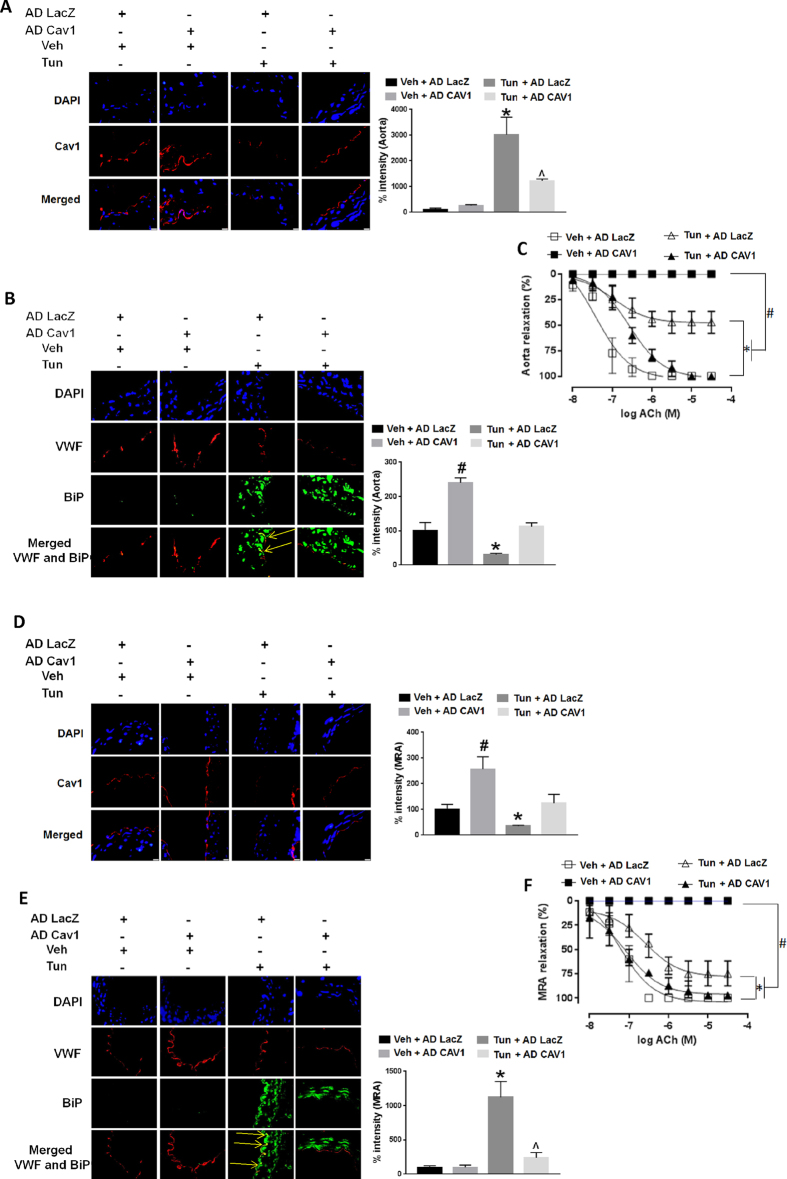
Cav1 rescues ER stress-induced endothelial dysfunction. Immunofluorescence for Cav1 with quantification on right (**A,D**), and ER stress marker (BiP) with quantification on right (**B,E**), and endothelium-dependent vasorelaxation (**C,F**) in thoracic aortas **(A,B,C)** and MRA **(D,E,F)**, of C57Bl/6 mice treated with tunicamycin (Tun) or vehicle control (Veh). and infected *ex vivo* with AD Cav1 or AD LacZ. *p < 0.05 between Tun + AD LacZ vs. Veh + AD LacZ, Veh + AD Cav1 and Tun + AD Cav1. ^#^p < 0.05 between Veh + AD Cav1 vs. Veh + AD LacZ and Tun + AD Cav1. ^^^p < 0.05 between Tun + AD Cav1 vs. Veh + AD Cav1 and Veh + AD LacZ. Yellow arrows point to BiP expression in the endothelium.

We then asked if downregulation of vascular Cav1 is responsible for endothelial dysfunction due to ER stress. Cav1 was reconstituted *ex-vivo* using AD Cav1 in aortas and MRA from mice treated with tunicamycin (Fig. 5A,D). Reconstitution of Cav1 mitigated vascular ER stress (BiP expression) in aortas and MRA (Fig. 5B,E), and partially rescued endothelium-dependent vasorelaxation in aortas and MRA of mice treated with tunicamycin (Fig. 5C,F). However, similar to our prior observation, overexpression of Cav1 in aortas and MRA from mice not treated with tunicamycin (Veh), led to marked impairment of endothelium-dependent vasorelaxation (Fig. 5C,F).

## Discussion

The present study sheds new light on the complex relationship between vascular Sirt1, miR-204, ER stress and Cav1 in the context of vascular endothelial function. Our data show that lack of Sirt1 is associated with ER stress and induction of miR-204, which is responsible for downregulation of Cav1. Downregulation of Cav1, in turn, contributes to impairment of endothelium-dependent vasorelaxation in both conductance and resistance arteries.

Sirt1 plays a salutary role in the endothelium by deacetylating eNOS, thus stimulating eNOS-derived NO^3^. Our data suggests that in addition to directly regulating eNOS activity, Sirt1 may also indirectly govern eNOS-derived NO by regulating the expression of modulators of eNOS activity such as Cav1. The role of Cav1 in vascular function is complex. On the one hand, Cav1 has been traditionally considered to suppress eNOS activation by binding to its calcium/calmodulin site and preventing its translocation and phosphorylation^17^,^18^. On the other hand, constitutive lack of Cav1 leads to super-physiologic levels of NO, with generation of secondary NO-derived reactive species such as peroxynitrite^18^, a culprit well-known to impair endothelial function. Thus, Sirt1-regulated Cav1 expression may achieve a sweet spot whereby Cav1 levels are sufficient to curtail NO production, while not excessive to suppress maintenance levels of NO. Supporting this contention is our experimental data that restoring Cav1 in aortas and MRA of mice subjected to ER stress rescued endothelium-dependent vasorelaxation, whereas overshooting Cav1 expression in vessels of mice not subjected to ER stress impaired endothelium-dependent vasorelaxation (Fig. 5A,C,D and F).

It is noteworthy that oxidative stress in mouse embryonic fibroblasts promotes sequestration of Sirt1 in caveolae through direct binding to Cav1, resulting in suppression of Sirt1 activity^5^. This finding, taken together with our data that Sirt1 is required for Cav1 expression, suggests a negative feedback loop for the fine tuning of Cav1 expression. As indicated above, such modulation of Cav1 expression may be especially important in the endothelium faced with oxidative stresses (such as ER stress) where, as our data suggests, either a significant decline or upregulation of Cav1 impair function.

Sirt1 expression is governed by microRNAs^7^. MiR-204 targets Sirt1 in non-vascular tissues^19^,^20^. In addition, recent evidence shows that miR-204 targets endothelial Sirt1, thereby leading to endothelial dysfunction^7^. Out data indicates that loss of Sirt1 in endothelial cells leads to upregulation of miR-204. Moreover, miR-204 functions as a mediator to suppress Cav1 expression in Sirt1 deficient/depleted endothelial cells and vascular tissue. Consistent with our results, miR-204 downregulates Cav1 in renal clear cell carcinoma^14^. Whether this suppression is direct or indirect is not clear, and will require further experimentation.

ER stress plays a key role in vascular endothelial dysfunction^16^. Moreover, several studies have reported a link between ER stress and decline of Sirt1 in other tissues^21^,^22^, though none have shown this in the vasculature. Additionally, our findings indicate that ER stress is associated with reduction in Cav1, and are in accordance with a study in human endothelial cells which showed that Cav1 is reduced in an ER stress–dependent manner^12^. Additional studies have shown that Cav1 overexpression rescues thapsigargin-induced ER stress and apoptosis in prostate cancer cells^13^ and support our findings relating to decrease in endothelial ER stress by overexpression of Cav1. Furthermore, studies show a role for miR-204 in the ER stress–responsive gene modulation and apoptosis in certain cancer cell types^9^,^10^ which is consistent with our findings that miR-204 plays a vital part in the ER stress response in endothelial cells.

**Figure 6:**
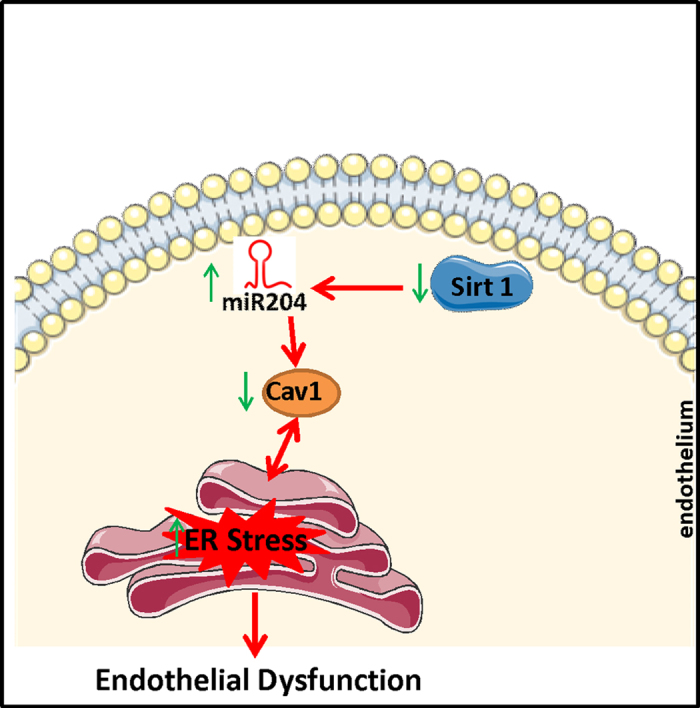
Schematic summarizing the main findings in this paper.

Finally, our data regarding the role of miR-204 in downregulating vascular Cav1 in a high-fat diet feeding model of obesity support studies showing that obesity in humans and in experimental models is associated with downregulation of Sirt1^23^,^24^,^25^,^26^ and Cav1^18^,^27^, and upregulation of miR-204^7^ in various tissues (Fig. 6).

## Research Design and Methods

### Animals

Experiments were performed on 8–12 week-old (1) C57Bl/6 mice and (2) mice with conditional deletion of endothelial Sirt1 (eSirt1^−/−^). The eSirt1^−/−^ mice were generated by crossing Sirt1^flx/flx^ mice with cadherin-5-Cre mice, in which the Cre recombinase is driven by an endothelium-specific cadherin-5 promoter. Sirt1^flx/flx^ mice were used as controls. Mice were fed standard chow (Research Diets Inc., New Brunswick, NJ) containing (in kilocalories) 10% fat, 70% carbohydrate, and 20% protein (D12450B). Terminal experiments were performed after mice were anesthetized (2–5% isoflurane). The thoracic aorta and the mesenteric resistance arteries (MRAs) were isolated and used for immunoblotting, immunostaining, *in situ* hybridization (ISH), real-time quantitative polymerase chain reaction (qPCR), and vascular reactivity. All protocols were approved by the Institutional Animal Care and Use Committee of the University of Iowa. All methods were performed in accordance with the guidelines and regulation of the University of Iowa.

### *In vivo* induction of ER stress by tunicamycin

Tunicamycin (Calbiochem, San Diego, CA USA) was dissolved in dimethyl sulfoxide (DMSO) and injected intraperitoneally at the dose of 0.75 mg/kg, two injections per week for 2 weeks into mice as previously described^28^. DMSO alone was used as vehicle in control mice.

### High fat (Western) diet

Mice were fed a HFD (Envigo, TD.88137) for 12 weeks. Mice fed with normal diet (ND) were used as controls.

### *In vivo* inhibition of miR-204

Locked nucleic acid miR-204 inhibitor (5′-AGG ATG ACA AAG GGA-3′; miR-204 I) or a scrambled nucleotide (5′-ACG TCT ATA CGC CCA-3′; miR SC) (Ambion Life Technologies, Grand Island, NY, USA) was systemically infused into mice using ALZET osmotic pumps that were implanted subcutaneously. Mice treated with tunicamycin were infused with miR-204 I or miR SC for 2 weeks (0.2 mg/week/mouse). HFD and ND mice were infused with miR-204 inhibitor or scrambled nucleotide for 6 weeks (0.7 mg/kg/day).

### *Ex vivo* infections with adenoviruses and transfections with a miR-204 inhibitor

MiR-204 I, miR SC, AD LacZ and AD Cav1 were transfected/infected into freshly isolated thoracic aortas and mesenteric resistance arteries (MRAs) from mice. Oligonucleotides were mixed with oligofectamine followed by addition to the medium. After 4 h, vascular rings were moved to fresh medium and further incubated for 24 h. Adenoviruses were directly added to the medium and incubated for 24 h.

### Mouse vascular reactivity

Male mice 8–12 weeks old were anesthetized and euthanized by rapid cardiac excision. The thoracic aorta and the MRAs were carefully harvested and placed in ice-cold Krebs buffer (118.3 mM NaCl, 4.7 mM KCl, 2.5 mM CaCl_2_, 1.2 mM KH_2_PO_4_, 25 mM NaHCO_3_, 1.2 mM MgSO_4_, 11 mM glucose, 0.0026 mM CaNa_2_ EDTA). Vessels were cleaned of fat and connective tissue, and cut transversely into 5–10 rings (1.8–2.0 mm wide). Vessels rings were placed in oxygenated chambers (95% O_2_/5% CO_2_) filled with 5 mL Krebs buffer solution and maintained at 37 °C and pH 7.4. Each ring was suspended between two wire stirrups in a 5 mL organ chamber of a four-chamber myograph system (DMT). One stirrup was connected to a three–dimensional micromanipulator and the other to a force transducer. The contractile force was recorded electronically. All rings were stretched to 2000 mg in 500 mg increments over a 1 h period to optimize the contractile response to KCl. One dose of KCl (60 mM) was added to verify vascular smooth muscle viability. Endothelium-dependent vasodilatation was determined by generating dose–response curves to acetylcholine (ACh) (10^−9^ to 3.10^−5^ M) in rings pre-constricted with phenylephrine (10^−6^ M) and was also expressed as percent maximal contraction.

### Immunostaining

Aortic and mesenteric arteries sections were de-paraffinized with xylene, followed by antigen retrieval by heating in citrate buffer (10 mM). Sections were probed with appropriate primary antibodies. Sirt1 antibody (Santa Cruz Biotech, Dallas, TX), Caveolin-1, Binding immunoglobulin protein (BiP), von Willebrand factor antibody (Abcam, Cambridge, MA) were used at a 1:50 to 1:200 dilution followed by a biotinylated secondary antibody for immunofluorescence. Sections were digitally imaged with an Olympus BX-61 microscope.

### Western blotting

Immunoblotting was performed in lysate from thoracic aorta and MRAs as previously described^29^. Chemiluminescent signal was developed using the Licor Odyssey Scanner (Lincoln, NE, USA). Bands were quantified using image J.

### *In situ* hybridization for miR-204

Aortic and MRA sections were de-paraffinized with xylene, followed by Proteinase K treatment (10 μg/mL for 5 min). ISH buffer (Exiqon, Vedbaek, Denmark; production #90000) was added with miR-204 probe (Exiqon, 5′-Dig-N-AGG CAT AGG ATG ACA AAG GGA A-N-Dig-3′) or with a scramble-miR probe (Exiqon, 5′-Dig-N-GTG TAA CAC GTC TAT ACG CCC A-N-Dig-3′) at 20 nM or 40 nM, and incubated for 72 h at 56 °C. After washing, the vessel sections were incubated in blocking solution for 15 min (5 mL PBS + 50 mg BSA + 100 uL Sheep serum + 2.5 uL Tween 20), followed by incubation with anti-DIG-FAB overnight (1:800 in antibody dilution solution). Slides were dipped in a solution containing BCIP/NBT (Roche, Mannheim, Germany) and incubated at 30 °C for 48 h. The slides were mounted with DPX and observed under the microscope.

### Cell culture, plasmid/siRNA transfections, and adenoviral infections

Human umbilical vein endothelial cells (HUVECs) were purchased from Clonetics (San Diego, CAUSA) and cultured in endothelial growth medium (EGM-2, Lonza, Walkersville, MD, USA). Cells were treated with tunicamycin (Tun, 1 μg/mL for 6 h). Cells were transfected with plasmids, miR-204 mimica (5′-UUC CCU UUG UCA UCC UAU GCC U-3′), miR-204 inhibitor (5′-AGG ATG ACA AAG GGA-3′) or scrambled miR (5′-ACG TCT ATA CGC CCA- 3′), validated siRNA-Sirt1, or negative control siRNA purchased from Invitrogen with Lipofectamine 2000 (Invitrogen, Carlsbad, CA). Cells were infected with 2 × 10^8^ viral particles per milliliter (VP/ml) of the control AD LacZ or the AD Cav1 and incubated at 37 °C for 24 h.

### QPCR

Total RNA was isolated by the acid guanidinium thiocyanate/phenol/chloroform method. Total RNA from cultured cells was isolated by the TRIZOL (Invitrogen) method. Real-time PCR was performed using the Prism 7000 Sequence Detection System (Applied Biosystems, Foster City, CA) with the SuperScript III Platinum SYBR Green One-Step qRT-PCR Kit (Invitrogen). The following primers (Exiqon) were used. Human GAPDH: forward 5′-ATG ACA TCAAGAAGGTGGTG-3′; reverse 5′-CATACCAGGAAATGAGCTTG-3′. Human Cav1 forward 5′-CTAATCCAAGCA TCCCTTTGCC-3′, reverse 5′-TTTATTACTGCCTCCTCCCCCA-3′. Human GAPDH (Quanta Biosciences, Beverly, MA, USA) was used as internal controls for mRNA quantification.

### Statistical analysis

Statistical analysis was performed using GraphPad Prism (Version 6.0) statistical software (GraphPad Software, Inc., San Diego, CA, USA). Significance of difference between two groups was evaluated using the *t*-test. For multiple comparisons, one-way analysis of variance (ANOVA) was used and *post-hoc* analysis was performed with Tukey’s test. Date are expressed as mean ± SEM and considered significant if *P-*values were ≤ 0.05. All shown data is representative of at least three independent experiments.

## Additional Information

**How to cite this article**: Kassan, M. *et al*. Sirtuin1 protects endothelial Caveolin-1 expression and preserves endothelial function via suppressing miR-204 and endoplasmic reticulum stress. *Sci. Rep.* **7**, 42265; doi: 10.1038/srep42265 (2017).

**Publisher's note:** Springer Nature remains neutral with regard to jurisdictional claims in published maps and institutional affiliations.
