# Corrigendum: Sirtuin1 protects endothelial Caveolin-1 expression and preserves endothelial function via suppressing miR-204 and endoplasmic reticulum stress

**DOI:** 10.1038/srep46123

**Published:** 2017-04-10

**Authors:** Modar Kassan, Ajit Vikram, Young-Rae Kim, Qiuxia Li, Adam Kassan, Hemal H. Patel, Santosh Kumar, Mohanad Gabani, Jing Liu, Julia S. Jacobs, Kaikobad Irani

Scientific Reports 7: Article number: 4226510.1038/srep42265; published online: 02 09 2017; updated: 04 10 2017

The original version of this Article contained errors in the spelling of the authors Modar Kassan, Ajit Vikram, Young-Rae Kim, Qiuxia Li, Adam Kassan, Hemal H. Patel, Santosh Kumar, Mohanad Gabani, Jing Liu, Julia S. Jacobs, Kaikobad Irani which were incorrectly given as M. Kassan, A. Vikram, Y. R. Kim, Q. Li, A. Kassan, H. H. Patel, S. Kumar, M. Gabani, J. Liu, J. S. Jacobs & K. Irani.

This has now been corrected in the PDF and HTML versions of the Article.

